# Molecular Mechanism of a Green-Shifted, pH-Dependent Red Fluorescent Protein mKate Variant

**DOI:** 10.1371/journal.pone.0023513

**Published:** 2011-08-22

**Authors:** Qi Wang, Laura J. Byrnes, Bo Shui, Ute F. Röhrig, Avtar Singh, Dmitriy M. Chudakov, Sergey Lukyanov, Warren R. Zipfel, Michael I. Kotlikoff, Holger Sondermann

**Affiliations:** 1 Department of Molecular Medicine, College of Veterinary Medicine, Cornell University, Ithaca, New York, United States of America; 2 Department of Biomedical Science, College of Veterinary Medicine, Cornell University, Ithaca, New York, United States of America; 3 Molecular Modeling Group, Ludwig Institute for Cancer Research and Swiss Institute of Bioinformatics, Lausanne, Switzerland; 4 Department of Biomedical Engineering, Cornell University, Ithaca, New York, United States of America; 5 Shemiakin-Ovchinnikov Institute of Bioorganic Chemistry, Moscow, Russia; 6 Nizhny Novgorod State Medical Academy, Nizhny Novgorod, Russia; Consejo Superior de Investigaciones Cientificas, Spain

## Abstract

Fluorescent proteins that can switch between distinct colors have contributed significantly to modern biomedical imaging technologies and molecular cell biology. Here we report the identification and biochemical analysis of a green-shifted red fluorescent protein variant GmKate, produced by the introduction of two mutations into mKate. Although the mutations decrease the overall brightness of the protein, GmKate is subject to pH-dependent, reversible green-to-red color conversion. At physiological pH, GmKate absorbs blue light (445 nm) and emits green fluorescence (525 nm). At pH above 9.0, GmKate absorbs 598 nm light and emits 646 nm, far-red fluorescence, similar to its sequence homolog mNeptune. Based on optical spectra and crystal structures of GmKate in its green and red states, the reversible color transition is attributed to the different protonation states of the *cis*-chromophore, an interpretation that was confirmed by quantum chemical calculations. Crystal structures reveal potential hydrogen bond networks around the chromophore that may facilitate the protonation switch, and indicate a molecular basis for the unusual bathochromic shift observed at high pH. This study provides mechanistic insights into the color tuning of mKate variants, which may aid the development of green-to-red color-convertible fluorescent sensors, and suggests GmKate as a prototype of genetically encoded pH sensors for biological studies.

## Introduction

Fluorescent proteins have facilitated major breakthroughs in modern molecular cell biology. They are widely used for studying biological events, such as cellular trafficking and endocytosis, and have contributed to the development of innovations such as multiphoton microscopy, genetically-encoded sensors, and super-resolution microscopy [1]–[3]. mKate, a derivative of eqFP578 from *Entacmaea quadricolor*, is a bright, monomeric, far-red fluorescent protein with excitation and emission peaks at 588 nm and 635 nm, respectively [4] (Table 1). These optical features of mKate provide important advantages for imaging in whole organism and multi-color labeling techniques [4], [7].

**Table 1:** Spectroscopic properties of mKate variants.

Protein	Mutations	Excitation max.(nm)	Emission max.(nm)	Ref.
mKate	-	588	635	[4], [5]
mKate2	V^48^A/S^158^A/K^238^A	588	633	[6]
mNeptune	M^41^G/S^61^C/S^158^C/Y^194^F	600	650	[7]
LSSmKate1	K^67^Y/P^127^T/S^143^G/M^159^E/T^176^S/M^189^V	463	624	[8], [9]
LSSmKate2	K^67^Y/P^127^T/S^143^G/M^159^D/T^176^S/M^189^V	460	605	[8], [9]
GmKate	S^143^C/S^158^A	455 (pH 5–9)/598 (pH 9–11)	525 (pH 5–9)/646 (pH 9–11)	This study.

Structural characterization of mKate under different conditions revealed the pH-dependent *trans-cis* isomerization of its chromophore [5], a discovery that further aided the development of mKate variants with enhanced optical properties (Table 1). Recently, two improved versions of mKate have been reported [6], [7]. mKate2 contains three mutations; mutation S^158^A destabilizes the trans-conformation, significantly enhancing mKate's brightness [5], [6], while mutations V^48^A and K^238^A accelerate protein maturation. Another, independent optimization yielded mNeptune with a peak excitation at 600 nm [7]. mNeptune is an auto-fluorescent protein with the greatest far-red-shift in excitation, and has displayed great potential in deep tissue imaging, as seen in living mice [7]. The shift in peak excitation was attributed to a collective effect from mutations S^158^C, M^41^G, S^61^C and Y^197^F. M^41^G provides space for a water molecule that forms a hydrogen bond with the acylimine oxygen of the chromophore. S^158^C enhances mNeptune's brightness following a similar mechanism as S^158^A in mKate2 [5], [6], but with much simplified photobleaching kinetics [7]. More recently, mKate variants with large Stokes shifts (LSS), LSSmKate1 and LSSmKate2, have been developed for multi-color, two-photon imaging [8]. An excited-state proton transfer model explains the nature of LSS, and provides insights in engineering novel red fluorescent proteins with LSS [9].

Although mKate and its variants (S^158^A mutant, S^158^C mutant and mNeptune) have been widely used as red-fluorescent proteins, they all contain a residual green fluorescent component when excited at 470 nm [7], while other mKate mutants exhibit significant green emission upon violet excitation [8]. Studies on orange and red fluorescent proteins suggested that mKate can undergo red-to-green photoconversion upon excitation at 405 nm and 561 nm [10], but the origin for the underlying red-to-green photoconversion mechanism is poorly understood. An understanding of the structural basis of green fluorescence in mKate variants will shed light on the necessary molecular interactions underlying two-color fluorescence, and may provide valuable insights aiding the development of enhanced color-switchable fluorescent proteins and biosensors.

Most of the well-understood color-switching mechanisms are based on studies of photoactivatable proteins. Irreversible photoconversion usually involves chemical modifications in or near the chromophore upon light irradiation and can be classified into two types. The type I mechanism involves a β-elimination reaction to further extend the chromophore's π-conjugation system, as observed for Kaede [11], EosFP [12], [13], IrisFP [14] (green to red transition), Dendra [15], and KikGR [16]. The type II mechanism, involving the decarboxylation of a glutamic acid residue near the chromophore, has been observed in PA-GFP [17], [18] PS-CFP [19] and PAmCherry [20]. Reversibly photoactivatable proteins may utilize a chromophore isomerization mechanism, as observed for IrisFP (dark to green, or dark to red transition) [14], asFP595 [21]–[24], rsTagRFP [25], KFP-HC [26], mTFP0.7 [27] and Dronpa [28]. Chromophore protonation and deprotonation also have been demonstrated to impact the optical properties of fluorescent proteins. Wild-type GFP has a bimodal absorbance spectrum with peaks at 395 and 475 nm, corresponding to the protonated chromophore and deprotonated chromophore, respectively [1]. The blue-to-green dual-color emission proteins deGFPs utilize a chromophore protonation switch mechanism in response to external pH changes [29]–[31]. Red fluorescent protein mKeima has a green emission component at 525 nm at very low temperature, and spectroscopic and structural studies illustrate that the green component stems from the protonated acylimine chromophore [32].

Protonation-dependent, ratiometric chromophores provide important advantages in pH imaging particularly in the context of *in vivo* imaging. pHluorin [33] exhibits dual excitation maxima in response to changes in pH, and has been used successfully in the study of synaptic activity and neuronal transmission [33], [34]. deGFPs [29]–[31] are a series of ratiometic pH indicators that have pH-dependent, blue-to-green dual emission properties, and are particularly useful for two-photon excitation [29]. In E^2^GFP, another pH sensitive probe, both excitation and emission fluorescence spectra are sensitive to changes in pH, allowing for the use of various light-excitation sources without affecting its linear response range [35]. However, a similar red-shifted sensor does not exist, in part due to the lack of a molecular mechanism regarding spectral tunability of the chromophore in mKate and related molecules.

While the spectral properties of the isolated chromophore of GFP has been the subject of many theoretical studies, fewer studies take into account the protein environment of the chromophore. However, recent hybrid quantum/classical (QM/MM) studies on, for example, DsRed.M1 [36], [37] and HcRed [23], [38] provide detailed insights into the protonation states of the chromophore and surrounding residues, as well as the spectrosopic properties of these red fluorescent proteins. Together, these studies highlight the potential of computational approaches to elucidate the molecular mechanism that contribute to spectral properties of fluorescent proteins and that are not always obvious from crystallographic and spectral analysis alone.

**Figure 1:**
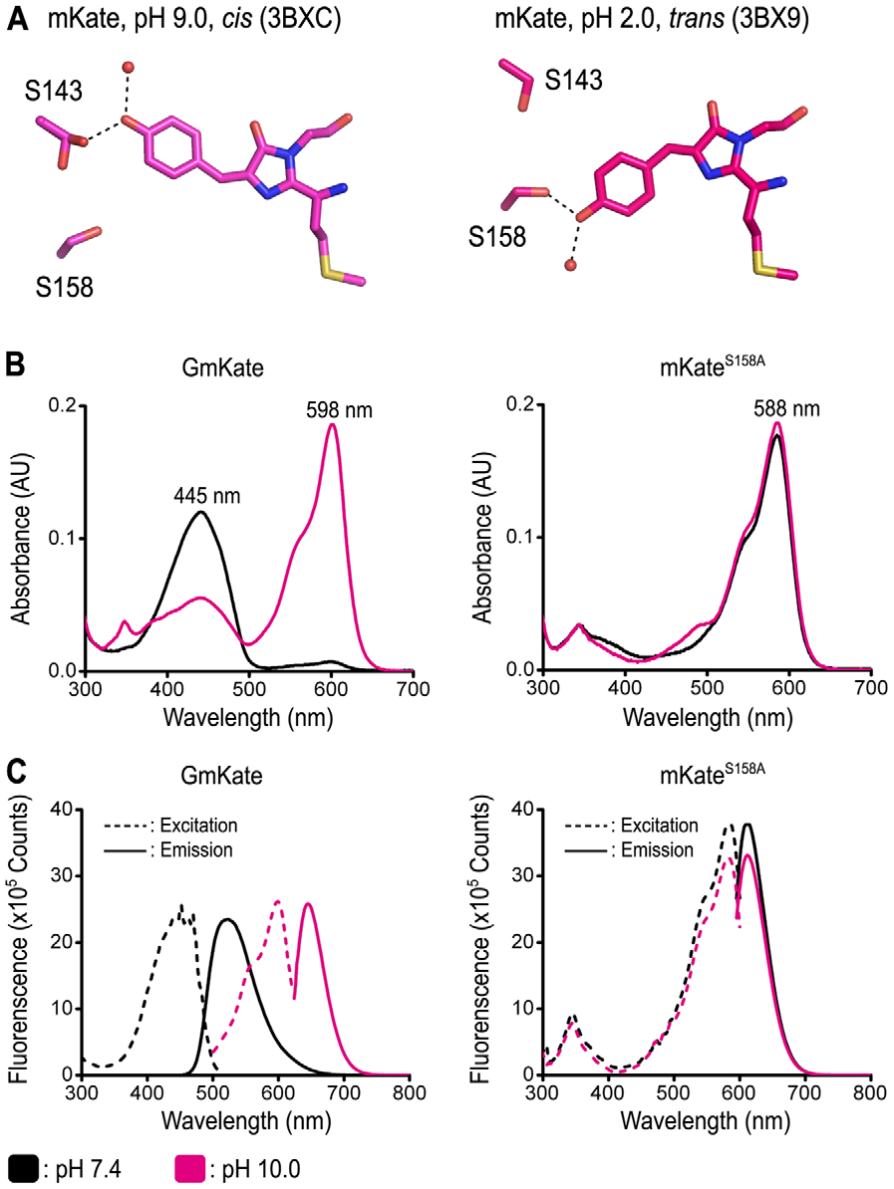
Spectroscopic properties of GmKate and mKate^S158A^. A. Position of S158 and S143 in the structures of mKate (PDB codes 3BXC and 3BX9) [5]. Only the chromophore and residues targeted for mutagenesis are shown for clarity. B. Absorbance spectra of GmKate and mKate^S158A^ at pH 7.4 and pH 10.0. The plots were scaled at the same protein concentration. Measurements were carried out at 25°C in buffer containing 150 mM NaCl, 25 mM HEPES or glycine. C. Excitation and emission spectra of GmKate and mKate^S158A^ at pH 7.4 and pH 10.0. Emission spectra for GmKate were recorded at an excitation wavelength of 445 nm (pH 7.4) and 598 nm (pH 10.0), respectively. Emission spectra for mKate^S158A^ were recorded at an excitation wavelength of 588 nm at both pHs.

Using a structure-informed approach, we introduced a minimal set of two mutations in the chromophore environment of mKate (Figure 1A). The first mutation, S^158^A, has been shown to destabilize the *trans* conformation of the chromophore [5]–[7], whereas the second mutation, S^143^C, was intended to impact the *cis* conformation. GmKate (mKate/S^158^A/S^143^C) displays distinct spectroscopic properties, most notably pH-dependent fluorescence with markedly increased green fluorescent component at physiological pH. Its optical properties are pH-dependent. Similar to the parent protein mKate, GmKate barely fluoresces at low pH (pH 2.0–4.0). At intermediate pH (pH 5.0–9.0), GmKate emits green fluorescence efficiently with absorbance and emission peaks at 445 nm and 525 nm, respectively. At high pH (pH 9.0–10.6), this variant appears blue under ambient light, with absorbance and emission maxima shifted to 598 nm and 646 nm, respectively. This color switching is fast and reversible, without a requirement of any external energy. Crystal structures revealed potential mechanisms for the color tuning and provide a molecular basis for the unusual bathochromic shift observed at high pH. Quantum chemical calculations confirm the attribution of the shift in absorbance to a change in protonation state of the chromophore.

## Results

### Optical spectra of GmKate

At pH 7.4, the absorbance spectrum of GmKate contains two maxima (Figure 1B). A major peak is located at 445 nm, which excites the 525 nm emission maxima (Figure 1C). The intensity of the minor peak with a maximum at 598 nm is about 10% of that of the major peak, and excitation at 598 nm yields minimal fluorescence (Figure S1A). The quantum yield of the 445/525 nm excitation/emission is 0.035 and the extinction coefficient is 19,500 M^−1^ cm^−1^ (Table 2), resulting in a brightness that is 6% of GFP and comparable to that of mGrape3 [7]. The green color emission is readily visible in protein solutions at ambient light (Figure S1B). In contrast, the single-mutant variant mKate^S158A^, which lacks the S^143^C mutation, has predominately one absorbance peak at 588 nm and emits strongly at 633 nm, giving the protein a visible red color [5], [6] (Figures 1B and 1C). The brightness of the single-mutant mKate^S143C^ is more than 100-fold less than that of mKate^S158A^ (Figures S2A and S2B), consistent with a destabilization of the *cis*-chromophore, which was predicted for this mutation based on the mKate crystal structure [5].

**Table 2:** Spectroscopic properties of GmKate.

GmKate	Excitation (nm)	Quantum yield	Extinction coefficient (M^−1^ cm^−1^)*
pH 4.0	445	0.0023	28,900
pH 7.4	445/598	0.035/0.065	19,500/2,130
pH 9.5	598	0.045	25,800

*: For reference, the extinction coefficient for EGFP is 50,000 M^−1^ cm^−1^.

**Figure 2:**
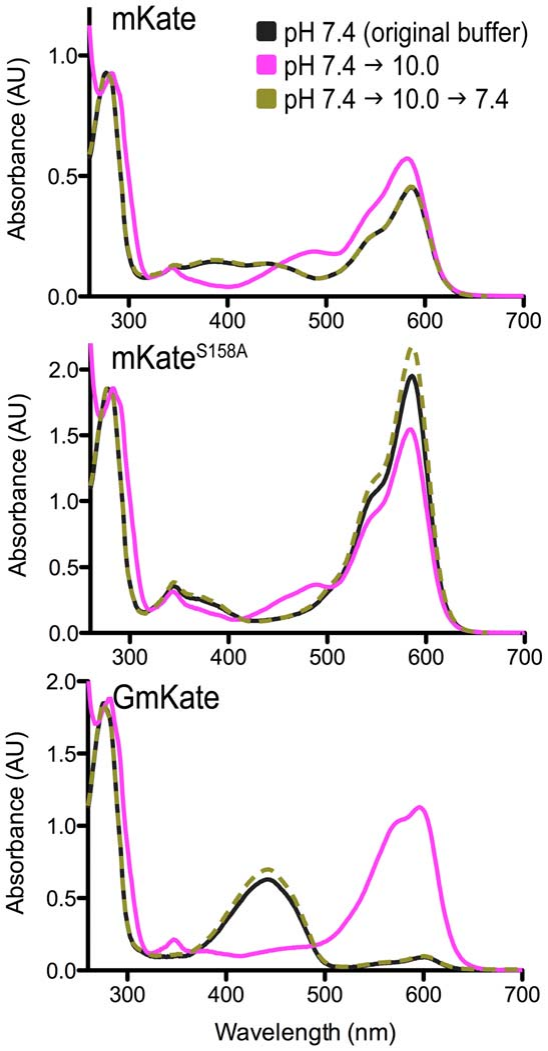
pH-dependent, reversible color tuning of GmKate. Absorbance spectra of GmKate, mKate^S158A^ and mKate at pH 7.4 and pH 10.0 are shown. The plots were scaled at the same protein concentration. Measurements were carried out at 25°C. Buffers with different pH were exchanged by using fast desalting columns.

Following acylimine hydrolysis [39], GmKate degrades into two fragments of apparent masses 13 and 20 kDa, corresponding to N-terminal and C-terminal segments spanning residues 1–63 and 64–223, respectively (Figure S3A). Essentially identical results were obtained for wild-type mKate. In contrast, EGFP remains intact as one major band in all of the experimental conditions tested here. These data indicate that the acylimine bond in GmKate has already matured, in contrast to the mechanism described for the green-color DsRed variant DsRed^K83R^ [39]. Therefore, it is unlikely that the observed green emission is due to a contribution of an immature chromophore. The reversible, pH-dependent tunability also supports this notion (Figure 2; see below).

At pH>9.0, solutions of GmKate turn blue in ambient light (Figure S1B), as has been described for mNeptune [7]. The absorbance peak at 598 nm increases about 13-fold, while the absorbance peak at 445 nm decreases to about half of that observed at pH 7.4 (Figure 1B). Excitation at 598 nm results in an emission peak at 646 nm (Figure 1C), about 13 nm red-shifted from wild-type mKate [4] and very close to the value reported for mNeptune (650 nm) [7]. The quantum yield of the 598/646 nm excitation/emission is also 0.045 and the extinction coefficient is 25,800 M^−1^ cm^−1^ (Table 2), resulting in a brightness that is 20% of mKate. The green-to-red transition induced by high pH is reversible (Figure 2) and occurs at a sub-second time scale. The transition is specific to the mKate double-mutant GmKate, and the fluorescence properties of the single-mutant mKate^S158A^ remain unchanged in the pH range 7.0–10.0, showing only a single 588/633 nm excitation/emission peak (Figures 1 and 2).

### Crystal structure of mKate^S143C^, mKate^S158A^ and GmKate at pH 7.4

The side chain of Serine 158 forms a hydrogen bond with the chromophore in the *trans* state [5]. Mutations in this position yield a brighter fluorescent protein with unaltered emission and excitation peak wavelengths [5]–[7]. Conversely, mutation of the side chain of Serine 143 removing its hydrogen bond to the *cis*-chromophore is predicted to destabilize the *cis*-conformation.

**Figure 3:**
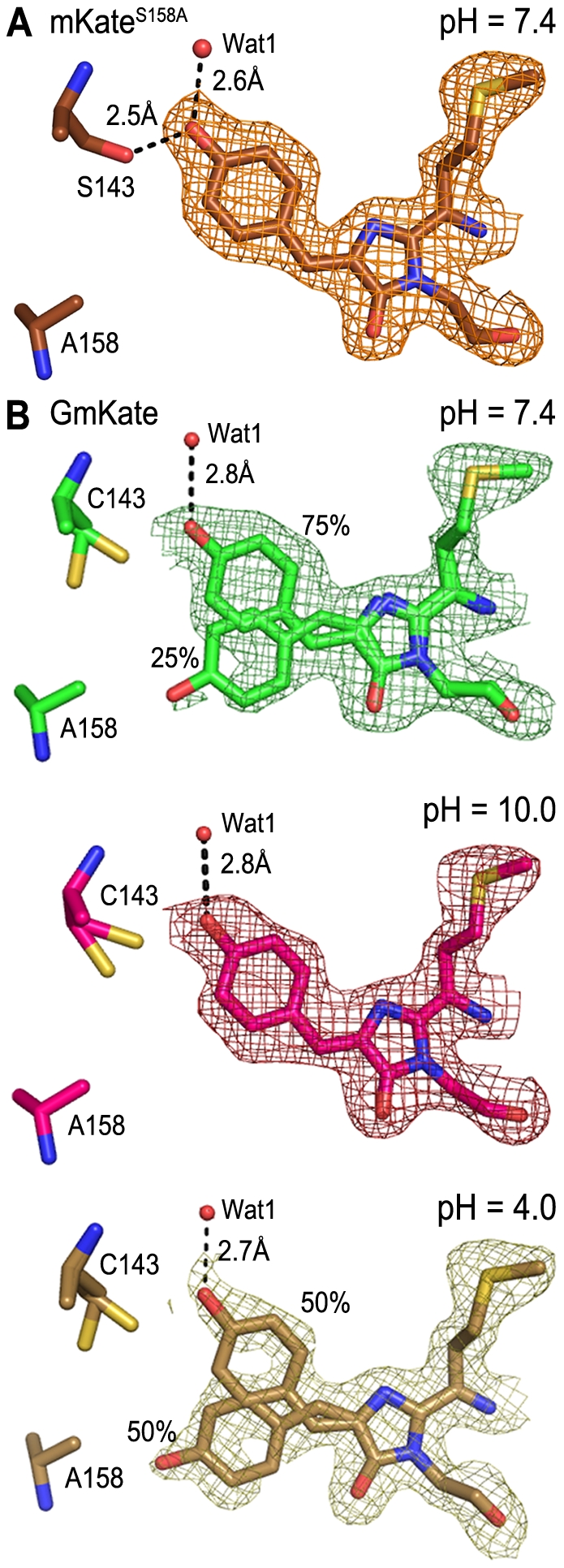
2Fo-Fc electron density maps of mKate^S158A^ and GmKate. A. Chromophore configurations of mKate^S158A^ at neutral pH. B. Chromophore configuration of GmKate at pH 7.4 (top panel), pH 10.0 (middle panel) and pH 4.0 (bottom panel). The density is contoured at 1.0σ for all maps. The occupancy of *cis*- and *trans*-chromophore was calculated during structure refinement.

**Figure 4:**
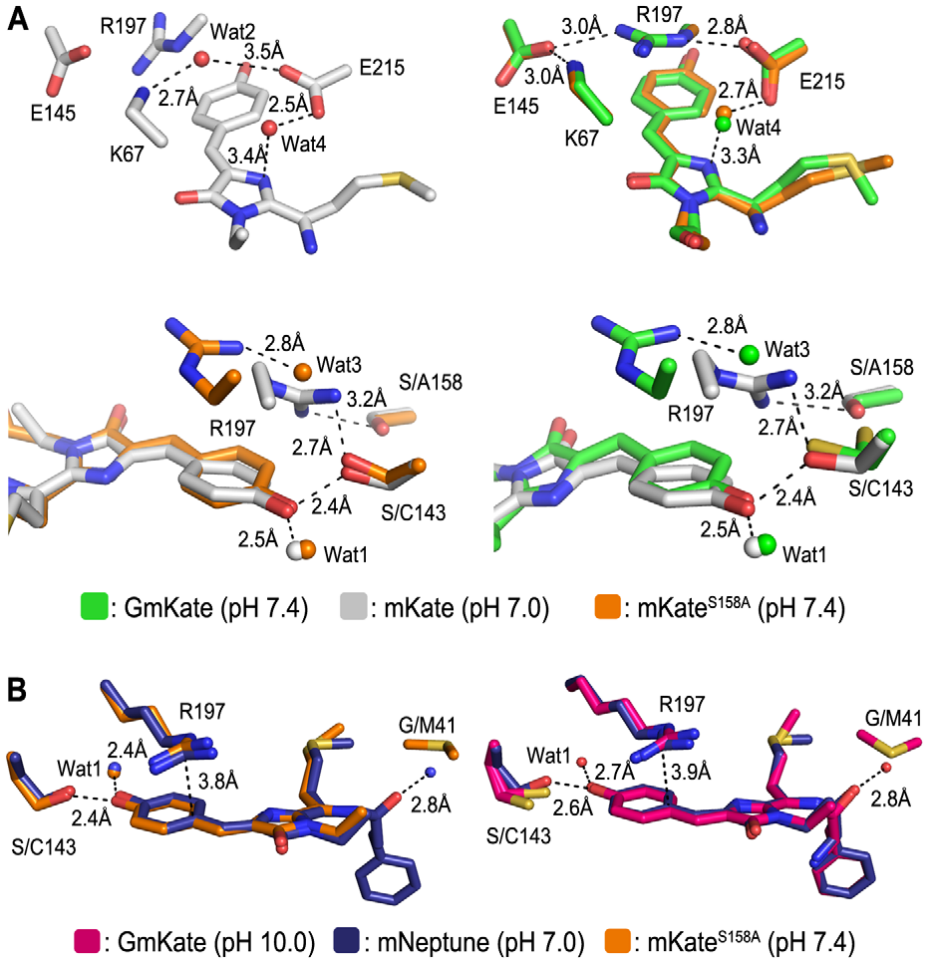
Structural comparison of mKate^S158A^, GmKate, mKate (PDB: 3BXB) and mNeptune (PDB: 3IP2). A. Superposition of mKate^S158A^ and GmKate with wild-type mKate [5] at neutral pH. B. Structural comparison of mKate^S158A^ and GmKate (pH = 10.0) with mNeptune [7]. Selected residues in vicinity of the chromophore are shown.

We crystallized the single point mutants to be used in a pairwise comparisons with GmKate (Table 3; see below). The chromophore in the crystal structure of mKate^S143C^ adopts only the *trans*-conformation at neutral pH, consistent with the proposed role of S^143^ in stabilizing the *cis*-conformation and the spectral properties of the protein (Figure S2). As predicted, the chromophore of mKate^S158A^ at pH 7.0 exists entirely in its *cis*-conformation (Figure 3A). The phenolate forms hydrogen bonds with S^143^ and a water molecule (Wat1) (Figure 3A), as observed in the structure of mKate at neutral pH [5]. In contrast to mKate structures, residues R^197^, E^215^ and several chromophore-coordinating water molecules undergo major structural rearrangements in mKate^S158A^ (Figures 4). R^197^ adopts a different rotamer conformation and forms hydrogen bonds with E^215^ and E^145^, while in mKate, R^197^ forms a hydrogen bond with S^143^ and S^158^. The space filled with the side chain of R^197^ in mKate^S158A^ is occupied by a water molecule (Wat2) in mKate, which forms a hydrogen bond with K^67^. These changes are generally comparable to those observed in mNeptune, which contains several other mutations in addition to S^158^C [7] (Figures 4A and 4B). Changes in the hydrogen bond network reveal a cavity in mKate^S158A^ (and GmKate) that is filled by a water molecule (Wat3) (Figure 4A). The water molecule is not present in the original mKate structure, and forms an additional hydrogen bond with R^197^ (Figure 4A).

**Table 3:** X-ray Data Colletion and Refinement Statistics.

	mKate^S158A^	mKate^S143C^	GmKate^pH4^	GmKate^pH7^	GmKate ^pH10^
Data collection^a^^,^ b					
X-ray source	CHESS A1	CHESS A1	CHESS A1	CHESS A1	CHESS A1
Wavelength (Å)	0.978	0.978	0.978	0.978	0.978
Space group	I4_1_	I4_1_	P2_1_2_1_2_1_	I4_1_	I4_1_
Unit cell parameters					
a, b, c (Å)	161.4,161.4, 76.2	161.6,161.6, 76.5	68.7, 102.0, 276.5	161.3, 161.3, 75.4	161.7, 161.7, 75.7
α, β, γ (°)	90, 90, 90	90, 90, 90	90, 90, 90	90, 90, 90	90, 90, 90
Resolution (Å)	50–1.90 (2.00–1.90)	50–2.70 (2.80–2.70)	50–1.74 (1.80–1.74)	50–1.90 (1.97–1.90)	50–1.98 (2.05–1.98)
No. of reflections					
Total	262264 (12546)	229839 (19617)	1553526 (112884)	653887 (45674)	565165 (35427)
Unique	73698 (3485)	26725 (2281)	199170 (19133)	73856 (6433)	64305 (5061)
Completeness (%)	95.5 (91.6)	98.0 (83.6)	99.4 (96.5)	97.9 (85.8)	95.2 (75.6)
Redundancy	3.6 (2.4)	8.6 (6.7)	7.8 (5.9)	8.9 (7.1)	8.8 (7.0)
*I*/σ(*I*)	11.0 (2.0)	12.9 (2.0)	20.3 (3.7)	20.6 (2.0)	17.8 (2.7)
*R*_meas_ (%)	6.9 (53.6)	11.2 (50.8)	8.9 (39.9)	9.4 (58.2)	10.0(40.9)
Refinement^c^					
*R_work_*/*R_free_* (%)	17.3/22.1	18.7/26.8	17.9/21.3	18.1/21.1	16.9/20.7
Bond r.m.s. deviations					
Length (Å)	0.007	0.009	0.006	0.007	0.006
Angles (°)	1.137	1.236	1.185	1.119	1.120
No. of atoms					
Protein	7250	7252	9531	7326	7245
Water	815	50	1969	712	736
Average B-factors (Å^2^)					
Protein	25.15	37.15	26.75	27.94	24.35
Water	29.18	35.55	31.97	33.66	30.65

a . Values as defined in HKL2000/SCALEPACK.

b . Highest resolution shell is shown in parenthesis.

c . Values as defined in PHENIX. All structures were solved by molecular replacement.

The guanidinium moiety of the arginine (R^197^) side chain contains a delocalized electron system, with the central carbon atom adopting a planar sp^2^ state. The particular conformation of R^197^ in mKate^S158A^ positions its guanidine group above the conjugated bond between the Y^62^ phenol ring and the imidazole moiety, presumably altering the conjugated π electron density distribution on the chromophore via a cation-π interaction (Figures 4A and S4A), as has been proposed for mNeptune [7].

In the crystal structure of green-fluorescent GmKate (pH 7.0) determined at 2.0 Å resolution, the chromophore adopts predominately the *cis*-conformation (75±5%; Figure 3A, top panel), which is comparable to the structure of the original mKate at pH 7.0 (80/20% *cis*/*trans*) [5]. Conformational changes of residues R^197^ and E^215^ as well as the position of chromophore-coordinating water molecules relative to mKate are similar to the rearrangements observed on mKate^S158A^ (Figure 4; see above). The absorbance peak at 445 nm in GmKate suggests that the chromophore resembles a neutral, DsRed-like chromophore or the A-form of LSSmKates [8], [9], though the chromophore and its surrounding residues largely resemble that of the red-fluorescent mKate^S158A^ (Figures 4A). The additional mutation in GmKate, S^143^C, creates a more hydrophobic environment, presumably impeding chromophore deprotonation. The chromophore is shifted 0.7 Å away from the position in mKate^S158A^, as measured by the distance between the phenolic oxygen atoms in the two structures (Figures 4A and S4B). This shift likely results from the loss of the hydrogen bond between the chromophore and S^143^ (a cysteine in GmKate), and/or the protonation of the phenolic oxygen moiety of the chromophore. Based on these observations, we propose that this neutral *cis*-chromophore is responsible for the green emission of GmKate.

LSS variants of mKate employ an excited-state proton transfer (ESPT) mechanism involving the side chains of S^158^ and D/E^160^ [9]. In GmKate, such an ESPT pathway may not exist since these key residues were subject to non-conservative changes to residues that do not support a hydrogen bond network with the chromophore (A^158^ and M^160^ in GmKate). Thus, our analysis suggests that the GmKate chromophore remains protonated at physiological pH, resulting in a neutralized chromophore that emits green fluorescence. This is in contrast to the mKeima ESPT-deficient mutant D^157^N, in which the mutation stabilizes the anionic state at physiological pH [32].

### Crystal structures of GmKate at pH 4 and 10

In the structure of far-red-emitting GmKate (pH 10.0, determined at 1.8 Å resolution), only the *cis*-conformation of the chromophore was observed (Figure 3B, middle panel). The absorbance peak at 598 nm is indicative of the anionic form of the chromophore, similar to the state observed in mNeptune [7]. Indeed, the π^+^-π stacking interaction between R^197^ and the chromophore is conserved at pH 10.0, and may further stabilize the deprotonated state of the chromophore (Figure 4B). Chromophore-interacting residues reside in positions similar to those observed at neutral pH. However, it is possible that these residues may carry different charges and differ in their protonation states, which is not apparent given the resolution of the crystal structures reported here.

The crystal structure of the dim state (pH 4.0, determined at 2.0 Å resolution) revealed equal occupancy of the trans- and cis-conformations, suggesting that the apparent pKa of *cis*-*trans* isomerization is close to 4.0 (Figure 3B, bottom panel). The *trans*-conformation is presumably a neutral (protonated) state, since the absorbance maximum remains unchanged at 445 nm in low pH buffers (Figure S1B). The loss of fluorescence may be attributed to the non-planar nature of the *trans*-conformation (Figure S4C) [5].

### Quantum chemical calculations of absorption spectra

In order to investigate the color tuning mechanism in our mKate variants, we carried out hybrid quantum/classical (QM/MM) and pure quantum chemical calculations for mKate^S158A^, GmKate^pH = 10^, and GmKate^pH = 7^. The geometry of the chromophore, R^92^, S/C^143^, R^197^, and Wat1, which is hydrogen-bonded to the phenolic oxygen of the chromophore, was optimized at the density functional theory level (PBE functional, TZVP basis set), taking the full protein environment into account at the classical level (CHARMM force field) (Figure S4D). From these optimized geometries we extracted different active-site clusters for excited state calculations. Since time-dependent DFT has been shown not to yield reliable results for similar chromophores [37], the ZINDO method was used as a computationally efficient alternative. As shown in Table 4, the calculated values accurately reproduce the experimentally observed shifts in absorption energy between the three protein structures, although the absolute values are blue shifted by about 0.25 eV. The green (pH 7) to red (pH 10) shift of the absorption energy of GmKate can indeed be attributed to the change in protonation state of the chromophore from neutral at pH 7 to anionic at pH 10. By comparing the absorption energies of the chromophore alone to their values in the protein, it is remarkable that the protein induces a large blue shift to the anionic chromophore (+0.17 eV in mKate^S158A^, +0.22 eV in GmKate^pH = 10^), while it leaves the neutral chromphore almost unaffected (+0.02 eV in GmKate^pH = 7^). About three quarters of the large blue shift can be attributed to interactions of the chromophore with R^92^, R^197^, E^215^, and a second water molecule (Wat4), while an additional blue shift is induced by interactions with Wat1, which is hydrogen bonded to the phenolic oxygen, and residue 143. The magnitude of this shift depends on the nature of residue 143; it is larger for serine (+0.06 eV), which can form an additional hydrogen bond, than for cysteine (+0.02 eV), which does not provide this possibility. Additional calculations showed that in case of the neutral chromophore, residues R^92^, S^143^, and Wat1 induce a blue shift of the absorption energy, while R^197^, E^215^, and Wat4 induce a red shift of about the same magnitude, so that the total protein influence approximately cancels out.

**Table 4:** ZINDO Absorption Energy Calculations.

	Exp.	Protein^a^	Chromophore^b^	Cluster 1^c^	Cluster 2^d^
mKate^S158A^	2.11 (588)	2.36 (526)	2.14	2.30	2.20
GmKate^pH10^	2.07 (598)	2.31 (536)	2.14	2.30	2.16
GmKate^pH7^	2.79 (445)	3.06 (405)	3.04	3.07	3.02

Energies are given in eV, wavelengths in nm in parentheses. All geometries were optimized within the protein environment using a QM/MM DFT approach.

a . Cluster including the chromophore, R^92^, S/C^143^, R^197^, E^215^, Wat1, and Wat4.

b . Anionic in mKate^S158A^ and GmKate^pH10^, neutral in GmKate^pH7^.

c . Cluster 1 includes the chromophore, R^92^, R^197^, E^215^, and Wat4.

d . Cluster 2 includes the chromophore, S/C^143^, and Wat1.

Taken together, these calculations corroborate the spectral and crystallographic data described above, and suggest that GmKate can be subjected to a bathochromic shift via a protonation/deprotonation switch.

## Discussion

### Structural basis of bathochromic shift in GmKate

In contrast to mKate^S158A^ and mKate, GmKate has a ∼10 nm red shift in absorbance and emission maxima at pH 10. A similar bathochromic shift has only been reported for mNeptune [7] (Table 1). The repositioning of R^197^ and E^215^ in GmKate relative to wild-type mKate cannot account for this shift since a similar rearrangement was observed in the crystal structure of mKate^S158A^ (Figures 3a and 3b). Based on the comparison of mKate^S158A^, GmKate^pH = 10^ and mNeptune structures, a potential mechanism for the observed red shift of GmKate at high pH can be proposed (Figure 3c). According to fragment molecular orbital calculations, chromophore emission is accompanied by changes in the charge distribution on the chromophore skeleton [40]. QM/MM and Symmetry Adapted Cluster/Configuration Interaction (SAC-CI) calculations [41] further suggest that a positive electrostatic potential near the phenolate moiety stabilizes the ground state of the chromophore and induces a blue shift to the electronic transition [42]. In the chromophore region, the only difference between mKate^S158A^ and GmKate^pH = 10^ is the loss of a hydrogen bond due to the substitution of the S^143^ with a cysteine (Figure 4A). This hydrogen bond may stabilize the highest occupied molecular orbital (HOMO) that has electron density concentrated on the phenolate motif [40]. The loss of this hydrogen bond would thus increase the HOMO energy, which results in a decreased energy gap between HOMO and the lowest unoccupied molecular orbital (LUMO), causing a red-shift. This interpretation is corroborated by our calculations (Table 4) and in line with a potential bathochromic shift mechanism for mNeptune, where the red shift is realized by a water molecule that interacts with the acylimine oxygen of the chromophore, stabilizing the LUMO [7]. Although R^197^ and E^215^ are not sufficient for the red-shift, the observation that their conformations are highly conserved among mKate^S158A^, mNeptune, and GmKate suggests that they play an important role during the illumination process, such as stabilizing the anionic *cis*-chromophore [7] and participating in proton relays (Figure S4E).

### Role of S^158^ and S^143^ in the *trans*-*cis* isomerization

Mutation S^158^A disrupts the hydrogen bond that stabilizes the *trans*-conformation and as a result the chromophore populates the *cis*-conformation in the mKate variants at physiological pH. Mutation S^143^C may de-stabilize the *cis*-conformation by removing a hydrogen bond between the side chain of S^143^ and the chromophore. In the crystal structures of the double-mutant, both *cis* and *trans* conformation are present to varying degrees, suggesting that while these two residues in mKate are involved in stabilizing the *cis* and *trans* state, they are not required for the isomerization. GmKate's reduced quantum yield relative to mKate [4], mKate^S158A^ [5], and mNeptune [7] suggests that its chromophore can be more flexible due to the lack of stabilizing hydrogen bonds. GmKate has a much higher apparent protonation pKa than mKate^S158A^ (Figure S3B), suggesting that S^143^ has an additional role in modulating the acidity of the chromophore. In GmKate, the S^143^C substitution establishes a more hydrophobic microenvironment, which may hinder the deprotonation of the chromophore. In addition to S^143^, residues R^197^ and E^215^ may also have an impact on the chromophore's pKa via electrostatic interactions.

### Reversible dark-bright and green-red, pH-dependent color switching mechanisms

**Figure 5:**
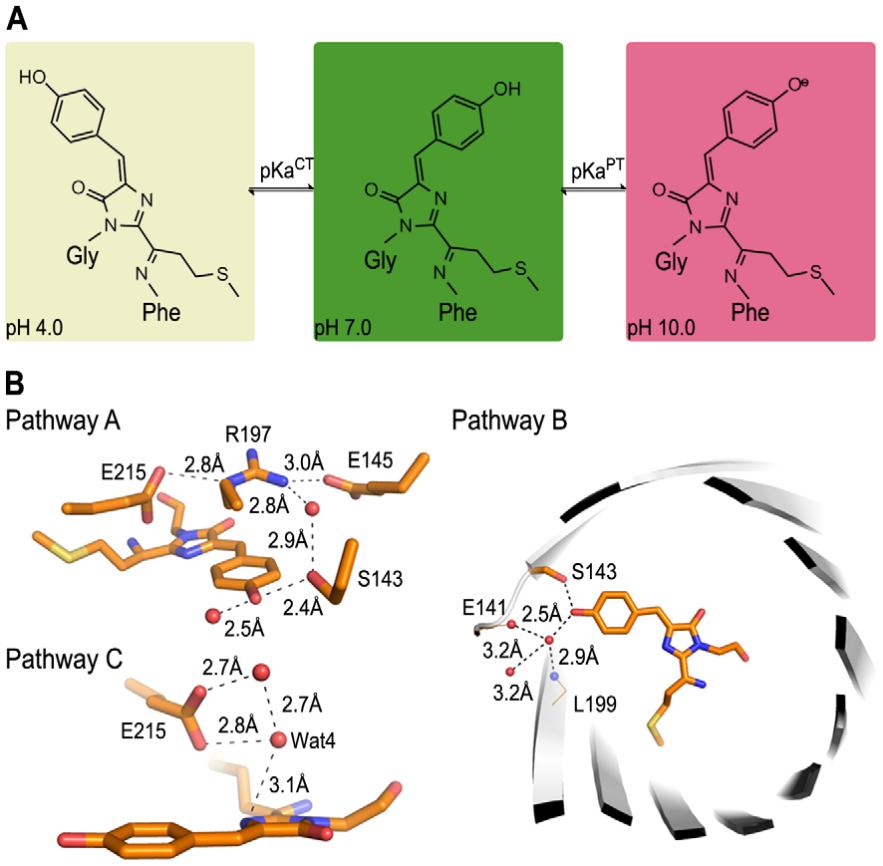
Coupled *cis-trans* isomerization/proton transfer model. A. Model for pH-dependent, reversible color-switching of GmKate. The chemical structures were drawn in ChemDraw. B. Putative proton relay pathways in mKate^S158A^. Three hydrogen bond networks are shown based on the crystal structure of mKate^S158A^.

The spectroscopic and structural analyses of GmKate in its dim, green and far-red states revealed a molecular mechanism of reversible green-red transition at an atomic level. The pH-dependence of GmKate involves two coupled processes: *trans-cis* isomerization and proton transfer (Figure 5A). Both processes are pH dependent, and we will use the symbols pKa^TC^ (*trans-cis*) and pKa^PT^ (proton transfer of *cis-*state) to denote the pH values at which the isomeric states are equally populated.

For GmKate, pKa^PT^ was determined to be 9.5±0.4, by fitting the 598 nm-absorbance pH titration profile with a first-order Hill equation (Figure S3B). pKa^TC^ was roughly estimated to be close to 4.0 based on the crystallographic data at pH 4.0 (Figure 3B). Since the optical analysis fails to distinguish between protonated *trans*- and *cis*-chromophores, we are not able to determine the pKa^TC^ using the pH titration assay. It should be noted that the pH-dependent *trans*-*cis* isomerization can be a multiple-step reaction, and that the pKa^TC^ therefore only presents the pH value at which *trans*- and *cis*-state are populated equally and cannot be used to estimate the *trans*/*cis*-ratio at other pHs. In addition, the observed pH value may not accurately reflect the pH values within the beta-barrel.

At a pH far above pKa^TC^, the majority of the chromophore resides in a *cis*-conformation, and it is the protonation state that determines the color of GmKate. At pKa^TC^<pH<pKa^PT^, the chromophore favors the neutral state, yielding absorbance/emission peaks at 445/525 nm, whereas in the pH range above the pKa^PT^, stabilization of the chromophore in the anionic state results in absorbance at 598 nm. The presence of two absorbance peaks for GmKate at 445 nm and 598 nm between pH 7.0–10.0 suggests that the chromophore is in equilibrium between the protonated and deprotonated state. This interpretation is also consistent with our observation that R^197^ adopts subtly different conformations in the four crystallographic protomers at high pH. ZINDO calculations demonstrate that the protein has a pronounced effect on the anionic chromophore, stabilizing its ground state by charged π-π interactions with the side chain of R^197^ and to a lesser extent by the hydrogen bond to R^92^. Interestingly the influence of these two residues on the absorption energy is not additive, meaning that the absence of just one of the two residues changes the calculated absorption energy of GmKate^pH = 10^ only little (−0.04 eV for R^197^, 0 eV for R^92^), while absence of both residues induces a large shift (−0.11 eV). Considering the collective results, we describe the color tuning mechanism in GmKate with a coupled *cis-trans* isomerization/proton transfer model (Figure 5A).

### Proton relay pathways in GmKate and mKate^S158A^

Unlike electrons that can undergo delocalization and quantum tunneling, proton transfer strictly requires proton donors and acceptors that are physically close together to form proton relay pathways, also known as proton wires [43]. The proton relay pathways that have been observed in LSSmKate1 and LSSmKate2 [9] do not exist in GmKate and mKate^S158A^. These two proteins utilize other entities to transport the proton during the deprotonation of the chromophore. The crystal structure of mKate^S158A^ reveals three potential proton wires (Figure 5B). In pathway A, the phenolic oxygen is in direct contact with the side chain of S^143^, which is hydrogen-bonded to R^197^ through a water-mediated interaction. R^197^ also interacts with E^145^ and E^215^ that can serve as proton acceptors. Such water mediated hydrogen-bond networks are frequently observed and have been shown to be responsible for ESPT in GFP and its variants [1]. In pathway B, the phenolic oxygen interacts with a water molecule that is coordinated by the backbone of E^141^ and L^199^. This water molecule is further hydrogen bonded to another crystallographically resolved water, which is located outside the ß-barrel. This pathway allows the direct proton transfer from the chromophore to the bulk solvent at high pH, which has also been observed for blue-green dual color emission of deGFPs [29]–[31] as well as other GFP variants [43], [44]. Alternatively but less likely for this variant given the observed geometries, in pathway C, the imine of the imidazole moiety may pass the proton to E^215^ through a water-mediated interaction, which has been described previously in asFP595 [23], [24]. For GmKate, only pathway B and C present viable mechanisms since pathway A relies on an intact S^143^, which has been mutated to cysteine in GmKate.

### Biological applications of GmKate

While the currently available pH-indicators based on GFP demonstrate the versatility and potential of genetically encoded pH sensors [29]–[31], [33]–[35], a similar probe based on mKate may have the advantage of non-overlapping spectra of the two states providing a higher dynamic range and possibly signal strength. The complex but tunable chemical configuration of the chromophore in mKate and its variants provides implications for the design of genetically encoded pH and other molecular sensors based on this molecule. In particular, our work suggests that the transition between red and green emission is realized through methods other than the application of external energy such as laser activation or covalent modification, which are usually irreversible and occur at a slower timescale. While the direct application of GmKate as a pH sensor is currently hindered by a non-physiologically high pKa and relatively low brightness compared to EGFP and mKate, further optimization via evolutionary mutagenesis and structure-based protein engineering may yield probes with improved properties.

Finally, a molecular principle similar to that identified for GmKate confers dim-to-bright transition to the genetically encoded calcium indicator GCaMP2, which contains a circularly permutated EGFP module [45]–[47]. A hydrogen bond network around the chromophore contributed to by the overall conformation of calcium-bound GCaMP2, stabilizes the deprotonated, bright state [46], [47]. In the absence of calcium, this network is broken, and the chromophore is in a protonated, dim state [46]. GmKate may provide an opportunity to create color-switching probes for cell-based and *in vivo* studies using GCaMP2 as a blueprint.

## Materials and Methods

### Protein expression and purification

The coding region corresponding to wild-type mKate ^1^ was amplified by standard PCR and cloned into the pRSET expression plasmid. GmKate, mKate^S143C^ and mKate^S158A^ plasmids were generated by using the QuikChange XL Mutagenesis Kit (Stratagene), following the manufacturer's instructions. GmKate used in the structural studies was cloned into a modified pET28a expression plasmid (Novagen) yielding an N-terminally hexahistidine SUMO fusion protein. The hexahistidine-tagged SUMO-moiety was cleavable using the protease Ulp-1 from *S. cerevisiae*.

*E.coli* BL21 (DE3) cells (Novagen) were transformed with plasmid DNA and grown in TB medium supplemented with 50 mg/l antibiotics at 37°C. At a cell density corresponding to an absorbance of 1.0 at 600 nm the temperature was reduced to 18°C, and protein production was induced by addition of 1 mM IPTG. Proteins were expressed for 12–16 hrs. Cells were collected by centrifugation, resuspended in NiNTA buffer A (25 mM Tris-Cl pH 8.2, 500 mM NaCl and 20 mM imidazole). After cell lysis by sonication, cell debris was removed by centrifugation at 40,000×g for 1 hr at 4°C. The clear lysate was loaded onto a HisTrap NiNTA column (GE Healthcare) equilibrated in NiNTA buffer A. The resin was washed with 20 column volumes NiNTA buffer A, and proteins were eluted in a single step with NiNTA buffer A supplemented with 500 mM imidazole. Fusion proteins were incubated with SUMO protease ULP-1 at 4°C overnight for removal of the hexahistidine-SUMO tag, and the cleaved protein was collected in the flow-through during NiNTA affinity chromatography. Proteins were further subjected to size exclusion chromatography on a Superdex200 column (16/60, GE Healthcare) equilibrated in gel filtration buffer (150 mM NaCl, 25 mM HEPES pH 7.4).

### Crystallization, X-ray data collection and structure refinement

Crystals were obtained by hanging drop vapor diffusion by mixing equal volumes of protein (∼20–50 mg/ml) and reservoir solution followed by incubation at 20°C. Crystals of mKate^S158A^ were obtained with the reservoir solution containing 0.1 M Tris-HCl pH 7.4, 0.25 M ammonium citrate dibasic and 22% PEG3350. Crystals of mKate^S143C^ were obtained with the reservoir solution containing 0.1 M Tris-HCl pH 7.4, 0.10 M ammonium citrate dibasic, and 18% PEG3350. GmKate crystals with an apparent green color were obtained with the reservoir solution containing 0.1 M Tris-HCl pH 7.0, 22% PEG3350, 0.1 M MgCl_2_. GmKate crystals appearing blue grew in 20% PEG3350, 0.1 M glycine pH 10.0, and 0.2 M MgCl_2_. The reservoir solution for crystallization of dim GmKate consisted of 18% PEG3350, 0.15 M DL-Malic Acid pH 4.0, 0.1 M sodium acetate trihydrate. All crystals were cryo-protected using crystallization solutions supplemented with 20% xylitol, frozen in liquid nitrogen, and kept at 100 K during data collection.

Data sets were collected using synchrotron radiation at the Cornell High Energy Synchrotron Source (CHESS, Ithaca, beamline A1, wavelength 0.977Å) (Table 3). Data reduction was carried out with the software package HKL2000 [48]. Phases were obtained from molecular replacement using the software package PHENIX [49] with the available structure of mKate (1.8 Å, pH 2.0, PDB code: 3BX9) [5] as the search model. Manual refinement in COOT [50] and minimization using PHENIX [49] yielded the final models with good geometry with all residues being in allowed regions of the respective Ramachandran plots. Illustrations were made in Pymol (DeLano Scientific).

### Absorbance and fluorescence spectroscopy

Absorbance spectra (260–800 nm, 10 mm path length) of purified proteins (20 µM) were recorded in duplicates on a DU730 UV/Vis spectrophotomer (Beckman Coulter) at 25°C. Emission spectra were recorded in triplicates on a fluorescence spectrophotomer (Photon Technology International) at a protein concentration of 100 nM and an excitation wavelength of 445 nm and 600 nm. Spectra at different pHs were measured by using buffers containing sodium phosphate (pH 2.0–4.0), sodium acetate (pH 4.5–5.0), MES (pH 5–6.5), HEPES (pH 6.5–8) or glycine (pH 8.5–10.5).

### Extinction coefficient calculation

Extinction coefficients were calculated according to the Beer's law. The absorbance maxima (445 nm and 598 nm) and protein concentrations were measured in duplicates on a DU730 UV/Vis spectrophotomer (Beckman Coulter).

### Quantum yield calculation

Quantum yields were calculated using Fluorescein, Rhodamine B and EGFP as optical standards. The absorbance maxima and the corresponding integrated emission intensities were measured for five different protein concentrations with the highest protein concentration yielding an optical density at 280 nm of ≤0.1. Although GmKate has dual excitation/emission peaks, only the predominant absorbance at a particular pH (445 nm for the green state, 600 nm for the red state) was considered. Linear fitting provided a slope value that is proportional to the quantum yield [51].

### Acrylimine hydrolysis assay

Proteins (2 mg/ml) were incubated in 15 µl gel filtration buffer, acidic buffer (0.1 M HCl) and alkaline buffer (0.1 M NaOH) for 10 min at room temperature [36]. Samples were incubated at 95°C for 10 min before loading on a 12% SDS-PAGE. The gels were stained with 1% Coomassie-blue R250 solution for 10 min and destained in 10% acetate acid solution overnight.

### Quantum chemical calculations

For each of the crystal structures of mKate^S158A^, GmKate^pH = 10^, and GmKate^pH = 7^, a CHARMM setup was generated, using the CHARMM22 force field [52] for the protein and the TIP3P model [53] for water. Hydrogen atoms were added using the HBUILD [54] function in CHARMM [55] at pH 7. The resulting system consisting of the protein and crystallographic water molecules was minimized in CHARMM by 100 steps of steepest decent using the GBMV2 implicit solvent model [56] and a restraint of 5 kcal/mol/Å^2^ on all heavy atoms towards their position in the crystal structure. QM/MM optimizations were performed with the Quickstep/FIST drivers [57], which are part of the freely available CP2K program (CP2K developers group; freely available from http://cp2k.berlios.de). Its QM/MM scheme is based on a multigrid technique for computing the electrostatic potential due to the MM atoms [58]. The QM part was treated with density functional theory using the Perdew-Burke-Ernzerhof function [59], Goedecker-Teter-Hutter pseudopotentials [60], a density cutoff of 280 Ry, and the TZV2P basis set, inside a cubic cell with an edge length of 30 Å. Geometry optimizations were carried out with the L-BFGS method [61] using standard convergence criteria, keeping the MM subsystem fixed. The QM region included the complete chromophore (M^63^-Y^64^-G^65^) as well as the backbone carbonyl and Cα atom of F^62^, the side chain of residue 143 up to the Cβ atom, the side chain of R^197^ up to the Cβ atom, and the water molecule hydrogen bonded to the phenol/phenolate oxygen (Wat1), resulting in a total number of 63/64 QM atoms. In mKate^S158A^ and GmKate^pH = 10^ the chromophore was described with a negatively charged phenolate, while in GmKate^pH = 7^ the chromophore was in its neutral form. Open valencies at the QM/MM border were saturated using capping hydrogen link atoms. Within CP2K it is possible to carry out QM/MM excited state calculations with time dependent density functional theory (TDDFT). However, it has been found repeatedly that TDDFT performs poorly for GFP chromophores [62]. Therefore we chose to extract a cluster from the optimized QM/MM structures consisting of the chromophore without its methionine side chain, plus the side chains of S/C^143^, R^92^, R^197^, E^215^, and the two water molecules within 3 Å of the chromophore (Wat1, Wat4). Absorption energies were calculated with Gaussian 09 [63] using the semi-empirical intermediate neglect of differential overlap for spectroscopy (INDO/S or ZINDO) method [64], which performs very well for the low-lying singlet states of organic chromophores and is widely used [65]. In Table 4 we report the vertical excitation energy of the lowest singlet state having a non-negligible oscillator strength. The influence of different residues on the absorption energy was probed by deleting them from the cluster calculations and recalculating the absorption energy.

#### Accession Numbers

Atomic coordinates and structure factors have been deposited in the RCSB Protein Data Bank under ID code 3SVU, 3SVS, 3SVR, 3SVN and 3SVO.

## Supporting Information

Figure S1 **Optical properties of GmKate.** A. Dual excitation and emission spectra of GmKate at pH 7.4. and pH 10.0. Measurements were carried out at 25°C in buffer containing 150 mM NaCl, 25 mM HEPES or glycine. B. Absorbance and excitation/emission spectra for GmKate at pH 4.0. C. GmKate in solution and in crystalline state at different pHs. Protein solutions were diluted in respective gel filtration buffers to a final concentration of 1 mg/ml.
(TIF)

Figure S2 **Spectroscopic and structural characterization of mKate^S143C^.** A. Absorbance spectra comparison between mKate^S143C^ and mKate^S158A^. Measurements were carried out at 25°C in buffer containing 150 mM NaCl, 25 mM HEPES, pH 7.4. B. Excitation and emission spectra comparisons between mKate^S158A^ and mKate^S143C^. C. 2Fo-Fc density map of the chromophore region. The map is contoured at 1.0σ. D. Structural comparison between wide-type mKate (PDB code 3BXB) [5] and mKate^S143C^.
(TIF)

Figure S3 **Biochemical characterization of GmKate.** A. Acrylimine hydrolysis assay. Samples were diluted into buffer containing 25 mM HEPES pH 7.5, 150 mM NaCl, and subjected to four different experimental conditions (A, no boiling; B, boiling; C, +0.1 M HCl; D, +0.1 M NaOH). Reactions were loaded onto a 12% SDS-PAGE followed by Coomassie staining. B. pH titration of Gmkate and mKate^S158A^. Absorbance at 598 nm was measured in triplicats in buffers ranging from pH 2.0–11.0. Apparent pKa values were calculated by fitting the titration curve to a first order Hill-equation in Origin 8.0.
(TIF)

Figure S4 **Structural characterization of GmKate.** A. A water-mediated hydrogen bond network surrounding the chromophore in GmKate (and mKate^S158A^) that is not present in mKate [5]. B. Superposition of GmKate with wild-type mKate at neutral pH (PDB code 3BXB). C. Structural comparison of a planar *cis* chromophore (GmKate, pH 10.0) and non-planar *trans* chromophore (GmKate, pH 4.0). D. Structural setup of the chromophore environment for quantum chemical calculations. The original crystal structures were superimposed and are shown with the carbon atomes colored in grey (rmsd over all atoms: mKate^S158A^, 0.108; GmKate pH 10, 0.120; GmKate pH 7.4, 0.144). E. A water-mediated hydrogen bond network in GmKate may facilitate chromophore protonation through π-π stacking and polar interactions.
(TIF)
