# Supplementary figures and images for: Molecular Mechanism of a Green-Shifted, pH-Dependent Red Fluorescent Protein mKate Variant
Source: PLoS One. 2011 Aug 22;6(8):e23513. doi: 10.1371/journal.pone.0023513 (PMC3161743; doi:10.1371/journal.pone.0023513)

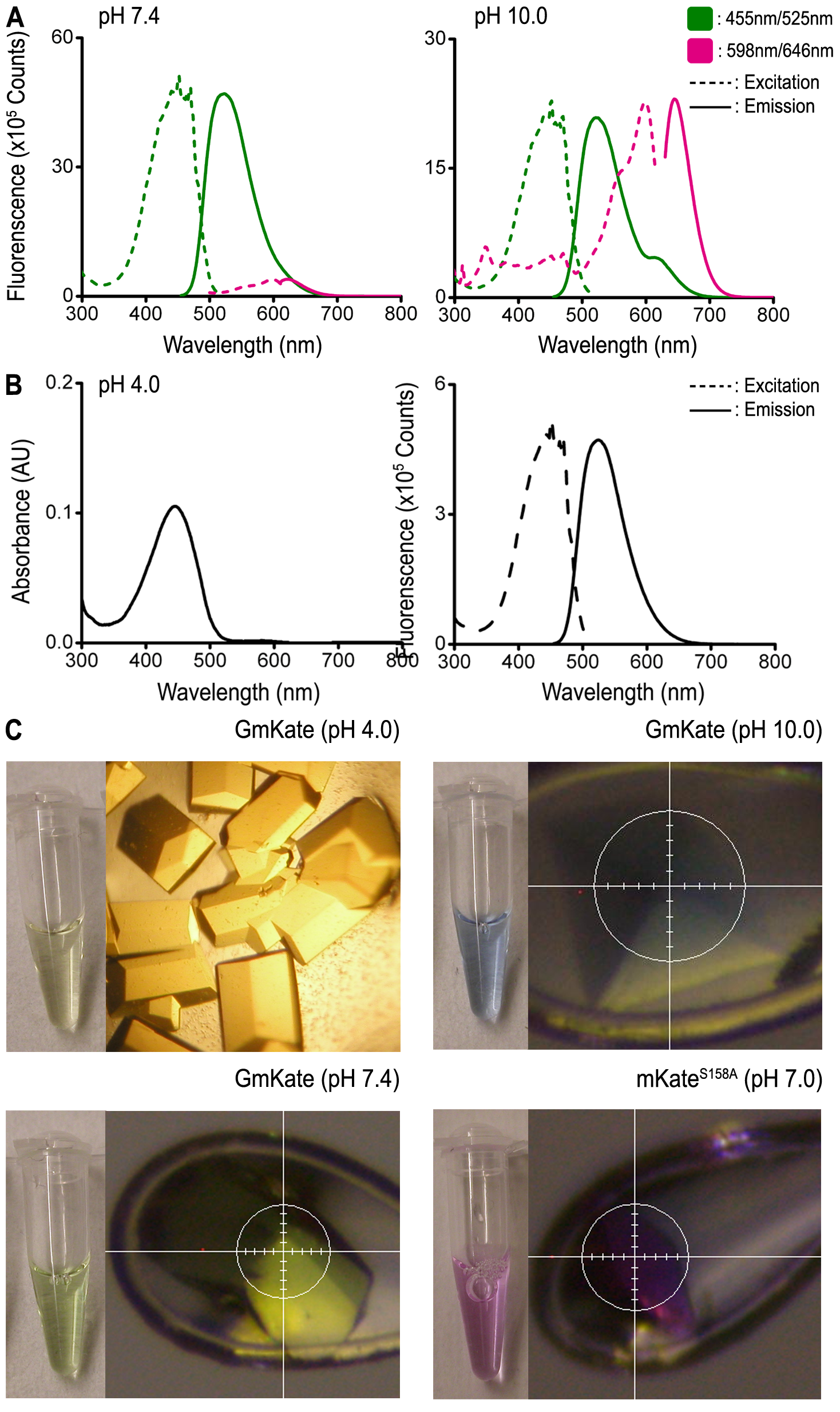

Supplement: Figure S1 — Optical properties of GmKate. A. Dual excitation and emission spectra of GmKate at pH 7.4. and pH 10.0. Measurements were carried out at 25°C in buffer containing 150 mM NaCl, 25 mM HEPES or glycine. B. Absorbance and excitation/emission spectra for GmKate at pH 4.0. C. GmKate in solution and in crystalline state at different pHs. Protein solutions were diluted in respective gel filtration buffers to a final concentration of 1 mg/ml. (TIF) [file pone.0023513.s001.tif]

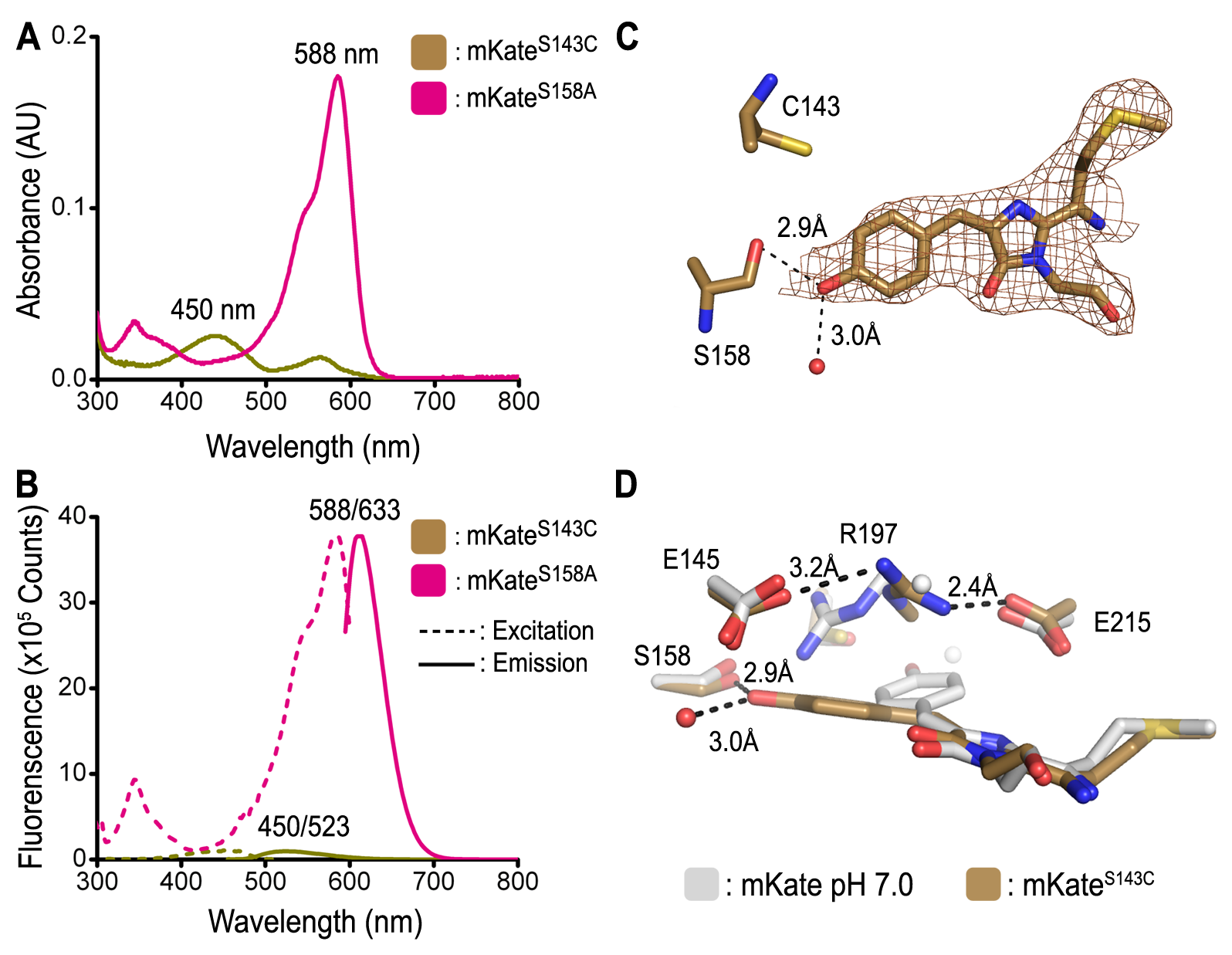

Supplement: Figure S2 — Spectroscopic and structural characterization of mKateS143C. A. Absorbance spectra comparison between mKateS143C and mKateS158A. Measurements were carried out at 25°C in buffer containing 150 mM NaCl, 25 mM HEPES, pH 7.4. B. Excitation and emission spectra comparisons between mKateS158A and mKateS143C. C. 2Fo-Fc density map of the chromophore region. The map is contoured at 1.0σ. D. Structural comparison between wide-type mKate (PDB code 3BXB) [5] and mKateS143C. (TIF) [file pone.0023513.s002.tif]

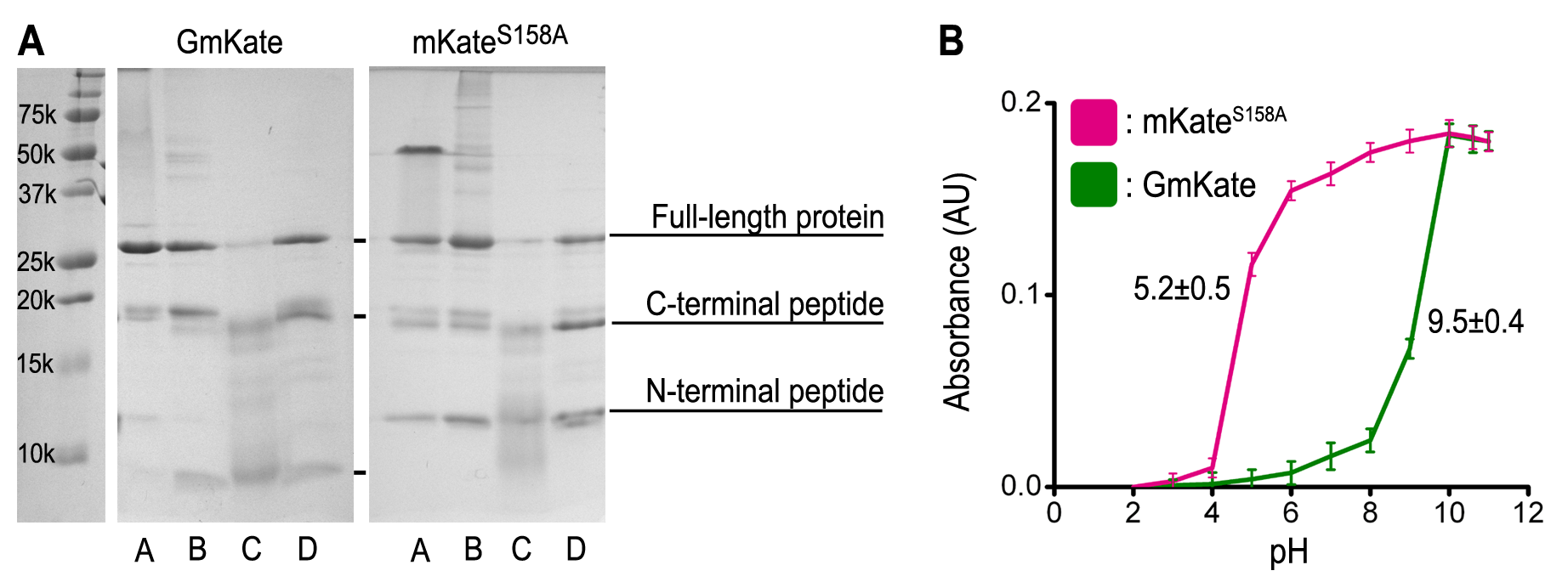

Supplement: Figure S3 — Biochemical characterization of GmKate. A. Acrylimine hydrolysis assay. Samples were diluted into buffer containing 25 mM HEPES pH 7.5, 150 mM NaCl, and subjected to four different experimental conditions (A, no boiling; B, boiling; C, +0.1 M HCl; D, +0.1 M NaOH). Reactions were loaded onto a 12% SDS-PAGE followed by Coomassie staining. B. pH titration of Gmkate and mKateS158A. Absorbance at 598 nm was measured in triplicats in buffers ranging from pH 2.0–11.0. Apparent pKa values were calculated by fitting the titration curve to a first order Hill-equation in Origin 8.0. (TIF) [file pone.0023513.s003.tif]

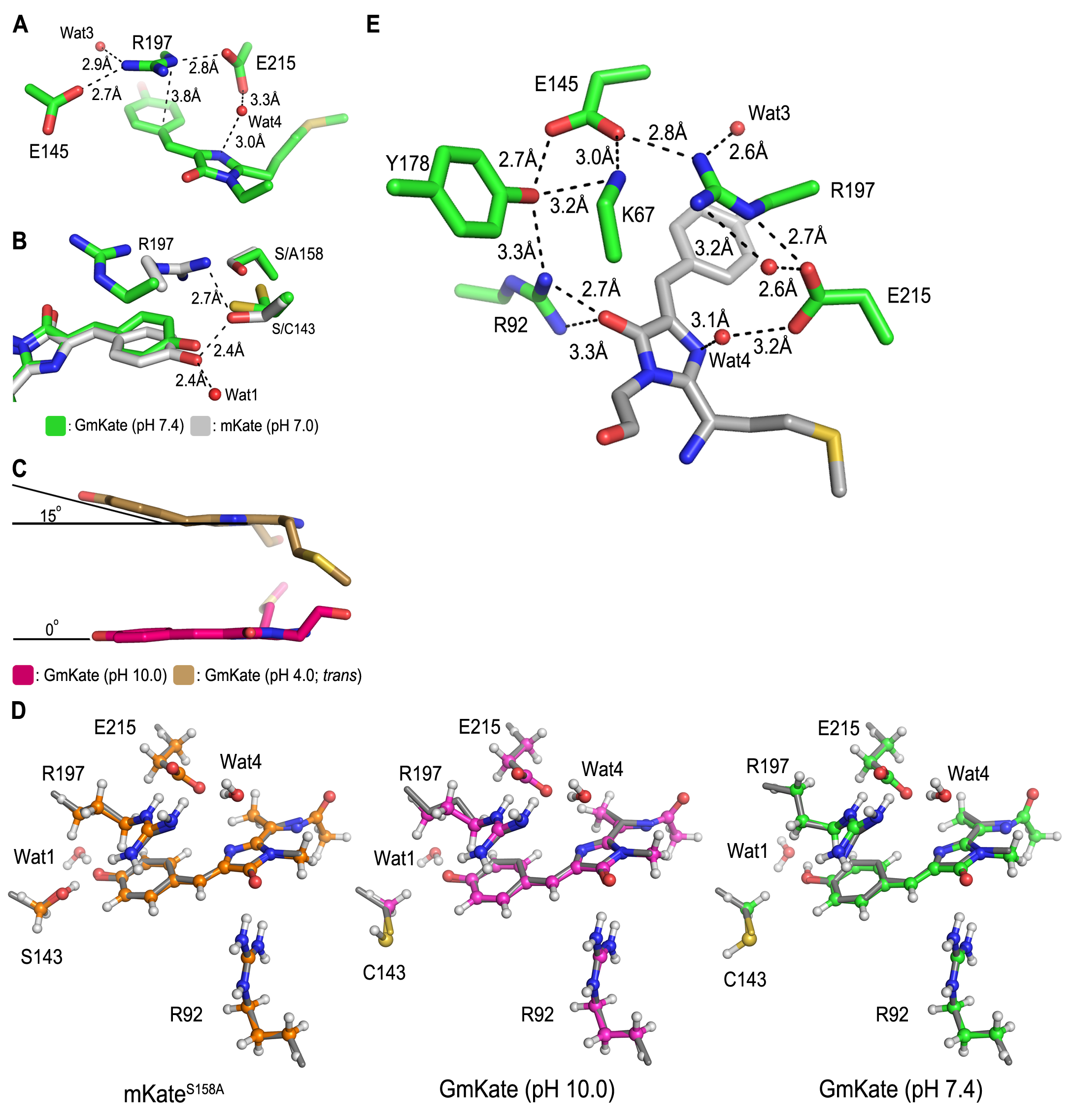

Supplement: Figure S4 — Structural characterization of GmKate. A. A water-mediated hydrogen bond network surrounding the chromophore in GmKate (and mKateS158A) that is not present in mKate [5]. B. Superposition of GmKate with wild-type mKate at neutral pH (PDB code 3BXB). C. Structural comparison of a planar cis chromophore (GmKate, pH 10.0) and non-planar trans chromophore (GmKate, pH 4.0). D. Structural setup of the chromophore environment for quantum chemical calculations. The original crystal structures were superimposed and are shown with the carbon atomes colored in grey (rmsd over all atoms: mKateS158A, 0.108; GmKate pH 10, 0.120; GmKate pH 7.4, 0.144). E. A water-mediated hydrogen bond network in GmKate may facilitate chromophore protonation through π-π stacking and polar interactions. (TIF) [file pone.0023513.s004.tif]
